# Genetic population of *Plasmodium knowlesi* during pre-malaria elimination in Thailand

**DOI:** 10.1186/s12936-021-03990-x

**Published:** 2021-12-03

**Authors:** Rungniran Sugaram, Patcharida Boondej, Suttipat Srisutham, Chanon Kunasol, Watcharee Pagornrat, Usa Boonyuen, Arjen M Dondorp, Aungkana Saejeng, Prayuth Sudathip, Mallika Imwong

**Affiliations:** 1 Division of Vector Borne Diseases, Department of Disease Control, Ministry of Public Health, Nonthaburi, Thailand; 2 Department of Clinical Microscopy, Faculty of Allied Health Sciences, Chulalongkorn University, Bangkok, Thailand; 3 Mahidol-Oxford Tropical Medicine Research Unit, Faculty of Tropical Medicine, Mahidol University, Bangkok, Thailand; 4 Department of Molecular Tropical Medicine and Genetics, Faculty of Tropical Medicine, Mahidol University, Bangkok, Thailand; 5 Centre for Tropical Medicine and Global Health, Nuffield Department of Medicine, University of Oxford, Oxford, UK

**Keywords:** *Plasmodium knowlesi*, *pkmsp1*, *pkdhfr*

## Abstract

**Background:**

Thailand is committed to eliminating malaria by 2024. From 2013 to 2020, the total number of malaria cases have decreased, from 37,741 to 4474 (an 88.1% reduction). However, infections with *Plasmodium knowlesi*, a monkey malarial pathogen that can also infect humans, have been increasingly observed. This study focused on the molecular analysis of *P. knowlesi* parasites causing malaria in Thailand.

**Methods:**

Under Thailand’s integrated Drug Efficacy Surveillance (iDES), which includes drug-resistance monitoring as part of routine case-based surveillance and responses, specimens were collected from malaria patients (n = 966) between 2018 and 2020. Thirty-one mono *P. knowlesi* infections (3.1%), most of which were from eastern and southern Thailand, were observed and confirmed by nested PCR assay and DNA sequencing. To evaluate whether these pathogens were from different lineages, cluster analysis based on seven microsatellite genotyping markers and the merozoite surface protein 1 (*pkmsp1*) gene was carried out. The *P. knowlesi* pyrimethamine resistance gene dihydrofolate reductase (*pkdhfr*) was sequenced and homology modelling was constructed.

**Results:**

The results of analysing the seven microsatellite markers and *pkmsp1* sequence demonstrated that *P. knowlesi* parasites from eastern Thailand were of the same lineage as those isolated in Cambodia, while the parasites causing malaria in southern Thailand were the same lineage as those isolated from Malaysia. The sequencing results for the *pkdhfr* genes indicated the presence of two mutations, Arg34Leu and a deletion at position 105. On analysis with homology modelling, the two mutations were not associated with anti-malarial drug resistance.

**Conclusions:**

This report compared the genetic populations of *P. knowlesi* parasites in Thailand from 2018 to 2020 and have shown similar lineages as those isolated in Cambodia and Malaysia of *P. knowlesi* infection in Thailand and demonstrated that the *P. knowlesi* parasites were of the same lineages as those isolated in Cambodia and Malaysia. The parasites were also shown to be sensitive to pyrimethamine.

## Background

Between 2010 and 2018, the incidence of malaria declined globally from 71 to 57 cases per 1000 head of at-risk populations. However, malaria still kills over 400,000 individuals every year [1]. In the Greater Mekong subregion (GMS), including Cambodia, China (Yunnan Province), Lao People’s Democratic Republic, Myanmar, Thailand, and Vietnam, the reported number of malaria cases fell by 76% between 2010 and 2018, and malaria deaths fell by 95% over the same period [1]. Thailand is committed to eliminating malaria by 2024. Between 2013 and 2020, the overall malaria incidence decreased from 37,741 to 4474 cases (88.1% reduction). The incidence of both *Plasmodium falciparum* and *Plasmodium vivax* malaria is declining, but the proportion of the two species has changed, with *P. falciparum* accounting for 5.7% (257/4474) of cases and *P. vivax* for 91.6% (4099/4474) in 2020 [2].

One of the *Plasmodium* species that infects humans, *Plasmodium knowlesi* [3], is a natural parasite of the long-tailed macaque, *Macaca fascicularis* and the pig-tailed macaque, *Macaca nemestrina*. Human malarial infection with this parasite was first reported in 1965 [4], and a second case presented in 1971 in Malaysia [5]. *Plasmodium knowlesi* infections have also shown distribution across the Greater Mekong Subregion (GMS), and previous *P. knowlesi* infections in the GMS have been recorded in Malaysia [6–9], Thailand [10–12], Myanmar [13, 14], Laos [15, 16], Vietnam [17, 18], and Cambodia [19]. The distribution of *P. knowlesi* may obstruct the malaria elimination agendas of countries of the GMS of Southeast Asia, especially due to asymptomatic cases, which have been previously reported [20].

In Thailand, the malaria information system set up by the National Malaria Control Programme (NMCP) of Thailand does not include information on *P. knowlesi* infections that occurred during the early stages of the system’s development; this is because the programme used Giemsa staining of thick and thin blood films and the pfHRPII-pLDH antigen rapid diagnostic test (pf-pan RDT) for diagnosis [21], which do not clearly distinguish *P. knowlesi* from other *Plasmodium* species. The NMCP of Thailand began using molecular techniques for confirmation as the most effective tool for malaria verification in quality control and quality assurance. *Plasmodium knowlesi* cases were subsequently detected in malaria patients who had visited the forest habitats of *M. fascicularis* and *M. nemestrina* macaques.

The present study aimed to analyse the genetic population of *P. knowlesi* parasites in Thailand and compare them with previous published findings of parasites isolated from Thailand [22, 23], Cambodia [20] and Malaysia [24–26]. Network analyses based on microsatellite markers were performed and constructed a phylogenetic tree based on the nucleotide sequences of the *P. knowlesi* merozoite surface protein 1 gene (*pkmsp1*). Furthermore, the *P. knowlesi* dihydrofolate reductase gene was isolated and analysed for mutations, and homology modelling of PkDHFR mutants was conducted.

## Methods

### Study sites and sample collection

Under the Thailand iDES, between 2018 and 2020, samples were collected from malaria patients (n = 966) to confirm *Plasmodium* species infection. The DNA samples were extracted using QIAmp DNA Mini Kit (Qiagen, Hilden, Germany) following the manufacturer’s instructions. Nested PCR based on the *18s rRNA* gene was performed following published protocol [27]. The amplified PCR product was purified using FavorPrep (Favorgen, Taiwan) and sent to Macrogen (South Korea) for DNA sequencing. Nucleotide and amino acid sequences of *18rRNA* were searched against the NCBI database using blastn (https://blast.ncbi.nlm.nih.gov/Blast.cgi).

### Molecular markers analysis

DNA samples were analysed for *P. knowlesi* microsatellite markers, NC03_2, CD05_06, NC09_1, NC10_1, CD11_157, NC12_2, and CD13_107, following a previous protocol [24]. Amplification of *pkmsp1* and full length *pkdhfr* was performed by nested PCR following previously published protocols [24]. The amplified PCR products of *pkmsp1* (covering nucleotides 4578 to 5376) and *pkdhfr* (covering nucleotides 1 to 708) were purified using the FavorPrep PCR purification kit. The purified PCR products of *pkmsp1* and *pkdhfr* were outsourced to Macrogen (South Korea) for sequencing. Nucleotide and amino acid sequences of these genes were aligned and compared with the reference sequences from *P. knowlesi* (accession no AM910987).

### Network analysis

The results of the analysis of *P. knowlesi* microsatellite genotyping markers were used in the cluster analysis using Network 10 software (https://www.fluxus engineering.com/sharenet.htm), which is based on Median Joining algorithms. Previously published *P. knowlesi* microsatellite data from asymptomatic infections from Cambodia (n = 8) [20], Peninsular Malaysia (n = 16), Sarawak, and Sabah (n = 22) and *P. knowlesi* infections from wild macaques (long-tailed and pig-tailed macaques) in Kapit (n = 18) [24] were combined for analysis to demonstrate the association between *P. knowlesi* parasites isolated from different parts of Thailand and those isolated from Cambodia and Malaysia.

### Phylogenetic tree analysis

To demonstrate the relationship between *P. knowlesi* parasites isolated in Thailand, Malaysia, and Cambodia, the DNA sequencing data of *P. knowlesi* merozoite surface protein 1 *(pkmsp1*) gene obtained in this study and previous data [22, 23, 25, 26]were used to construct a phylogenetic tree, using MEGA X (https://www.megasoftware.net/) based on neighbour-join (NJ) and BioNJ algorithms with branch lengths measured as the number of substitutions per site.

### Homology modelling of PkDHFR mutants

Homology models of the wild type (WT) PkDHFR and two mutant (Arg34Leu and Thr105 deletions) proteins in complex with the inhibitor (pyrimethamine) were constructed, using SWISS-MODEL server (https://swissmodel.expasy.org/interactive) based on the x-ray structure of PvDHFR at a resolution of 1.90 Å (PDB ID: 2BL9) [28]. The models were validated by PROCHECK [29].

## Results

### *Plasmodium knowlesi* infection in Thailand

**Fig. 1:**
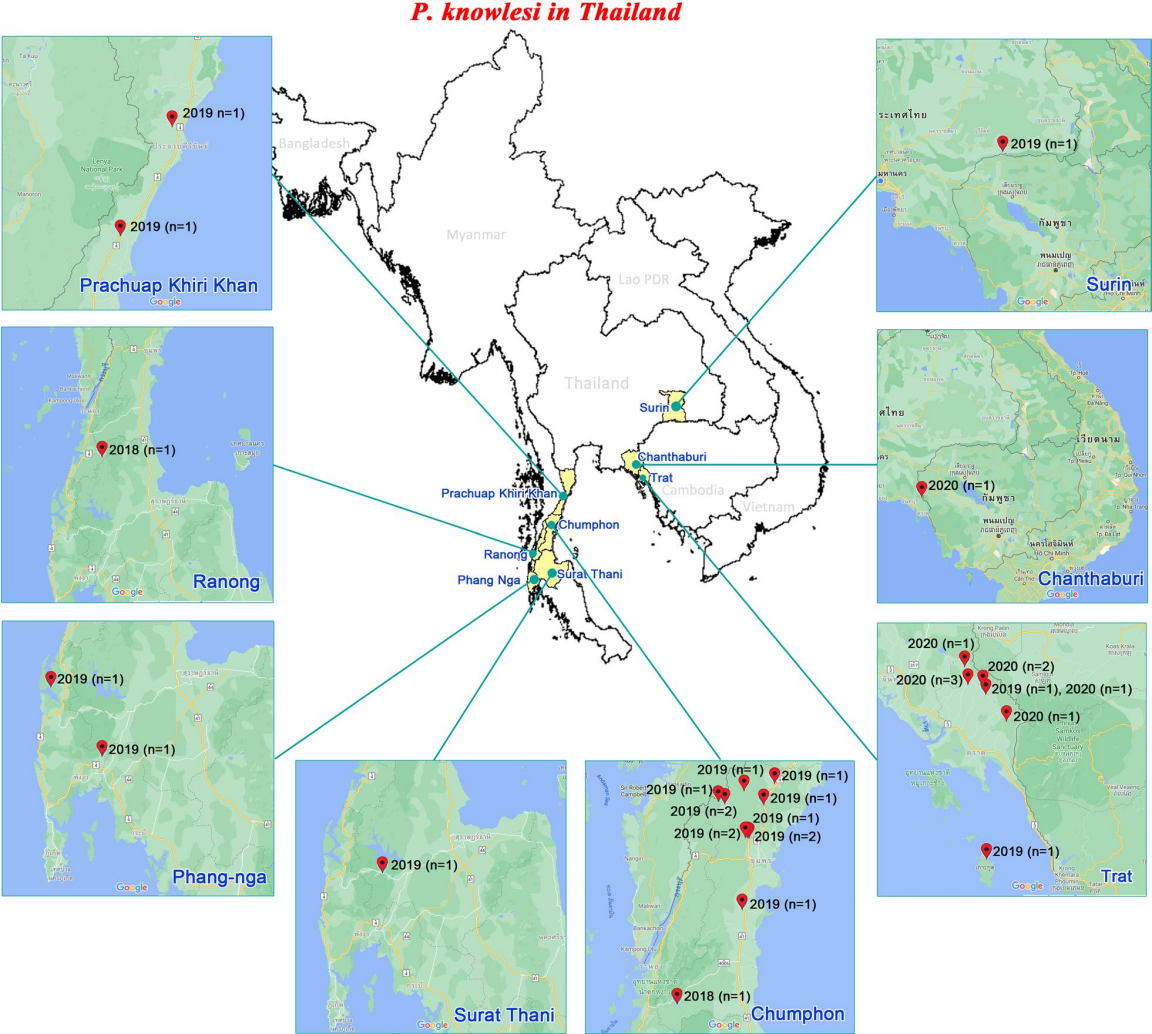
Study sites of specimen collection for the study

Of the 966 malaria collected samples, 31 (3.2%) mono-infections with *P. knowlesi* were confirmed by nested PCR assay and DNA sequencing. The samples collected from eastern Thailand included isolates from Surin (n = 1), Chanthaburi (n = 1), Trat (n = 10), and those from southern Thailand included isolates from Prachuap Khiri Khan (n = 2), Chumphon (n = 13), Ranong (n = 1), Surat Thani (n = 1), and Phang-nga (n = 2) in 2018 and 2019 (Fig. 1; Table 1).

**Table 1:** Collected samples from iDES for PCR species confirmation

	PF	PV	PO	PM	PK	Mixed PF+PV	Total
2018	29	139	0	0	2	5	175
2019	50	289	0	14	20	2	375
2020	24	369	0	12	9	2	416
Total	103	797	0	26	31	9	966

### Diversity and network analysis of microsatellite and *pkmsp1* genotyping

**Fig. 2:**
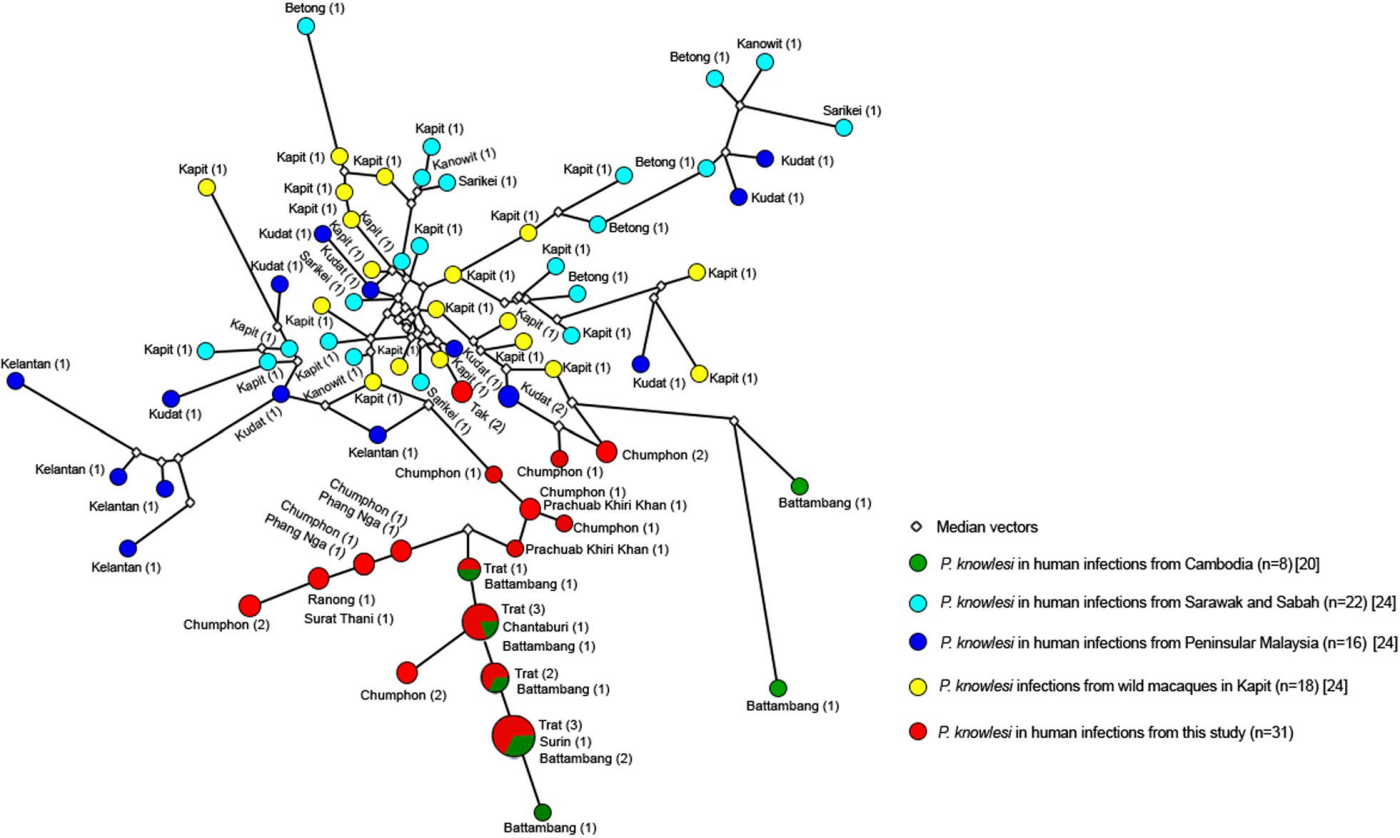
Network analysis based on microsatellite markers. Previous published findings were obtained from [20, 24]

To evaluate the lineage relationships among these *P. knowlesi* infections, microsatellite genotyping and cluster analyses were performed. The overall mean heterozygosity was relatively low (He = 0.327, SE = 0.043), the mean number of alleles was 3.1, and the multiplicity of infection was 1.032. No significant difference in microsatellite genotypic diversity was found between samples from eastern and southern Thailand. Haplotype network analysis was performed with the microsatellite marker results (Fig. 2) and showed that *P. knowlesi* isolated from eastern parts of Thailand, including Surin, Chanthaburi, and Trat, were the same haplotype as *P. knowlesi* parasites isolated from Battambang, Cambodia. Contrastingly, most of the *P. knowlesi* parasites isolated from southern parts of Thailand, (Prachuap Khiri Khan, Chumphon, Ranong, Surat Thani, and Phang-nga) were in the same lineage as the parasites isolated from Malaysia [24].

**Fig. 3:**
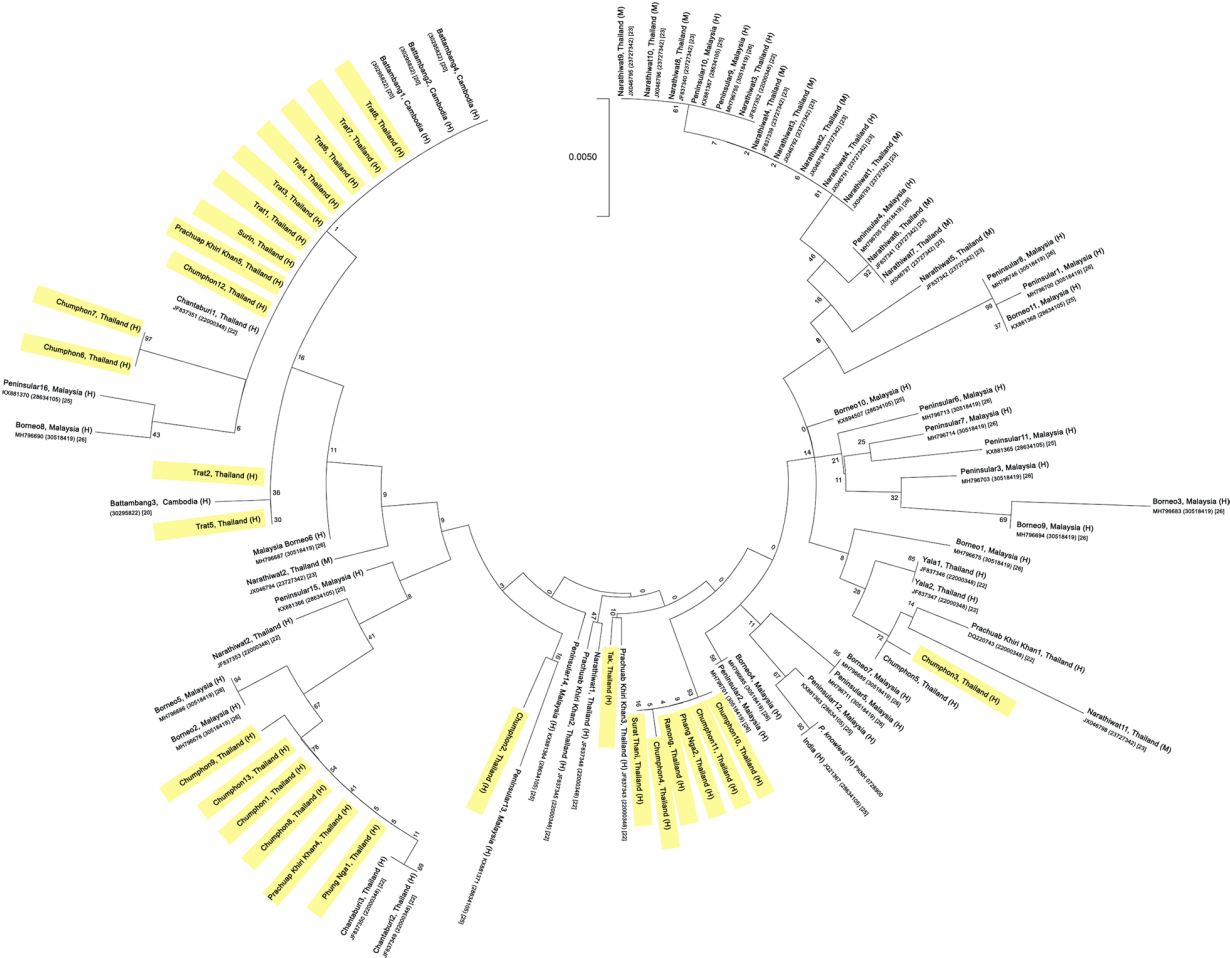
Phylogenetic tree analysis based on *pkmsp1* gene. The data obtained from this study are highlighted in yellow. M represents *pkmsp1* from wild macaques, H represents *pkmsp1* from human infection. Previous published findings were obtained from [20, 22, 23, 25, 26]

*Plasmodium knowlesi* samples from Thailand were used to create a dendrogram of the merozoite surface protein 1 (pkmsp1), which was compared to previous findings from Thailand [22, 23], and Malaysia [25, 26] was developed (Fig. 3). *Plasmodium knowlesi* samples collected from southern Thailand, including those connected by nodes, represent descendants from a common ancestor and are more genetically similar to the *P. knowlesi* isolates from Malaysia; while *P. knowlesi* isolates from eastern Thailand showed high similarity with *P. knowlesi* isolates from Cambodia. Moreover, the malaria isolated from Tak province are closely related to those isolated from Prachuap Khiri Khan.

### *Pkdhfr***gene analysis**

Full-length *pkdhfr* DNA sequences were amplified successfully from 16 isolates and were aligned with the reference sequence from *P. knowlesi* strain H (PKNH_0509600) to investigate the variations of the gene. The five mutations in the biding pocket of PkDHFR, equivalent to *P. vivax* DHFR (I13, F57, S58, S117, I173), were not observed in this study. However, two mutations were found, including Arg34Leu (11/16) and a three-nucleotide deletion at Thr105 (5/16). The PkDHFR mutation at Arg34Leu is equivalent to that in PvDHFR at Arg34. Although it was an amino acid deletion at Thr105, it did not affect the reading frame and resulted in no premature termination. This position is equivalent to the tandem repeat regions (amino acids 88 and 103 GGDNTS) in PvDHFR, which have been observed previously [30]. The Arg34Leu mutation was found in the isolates from southern Thailand, while the Thr105 deletion was found in isolates from eastern Thailand, which is close to Cambodia. The Thr105 deletion was also found in all *P. knowlesi* isolates (n = 8) from Cambodia.

### Homology modelling of PkDHFR mutants

**Fig. 4:**
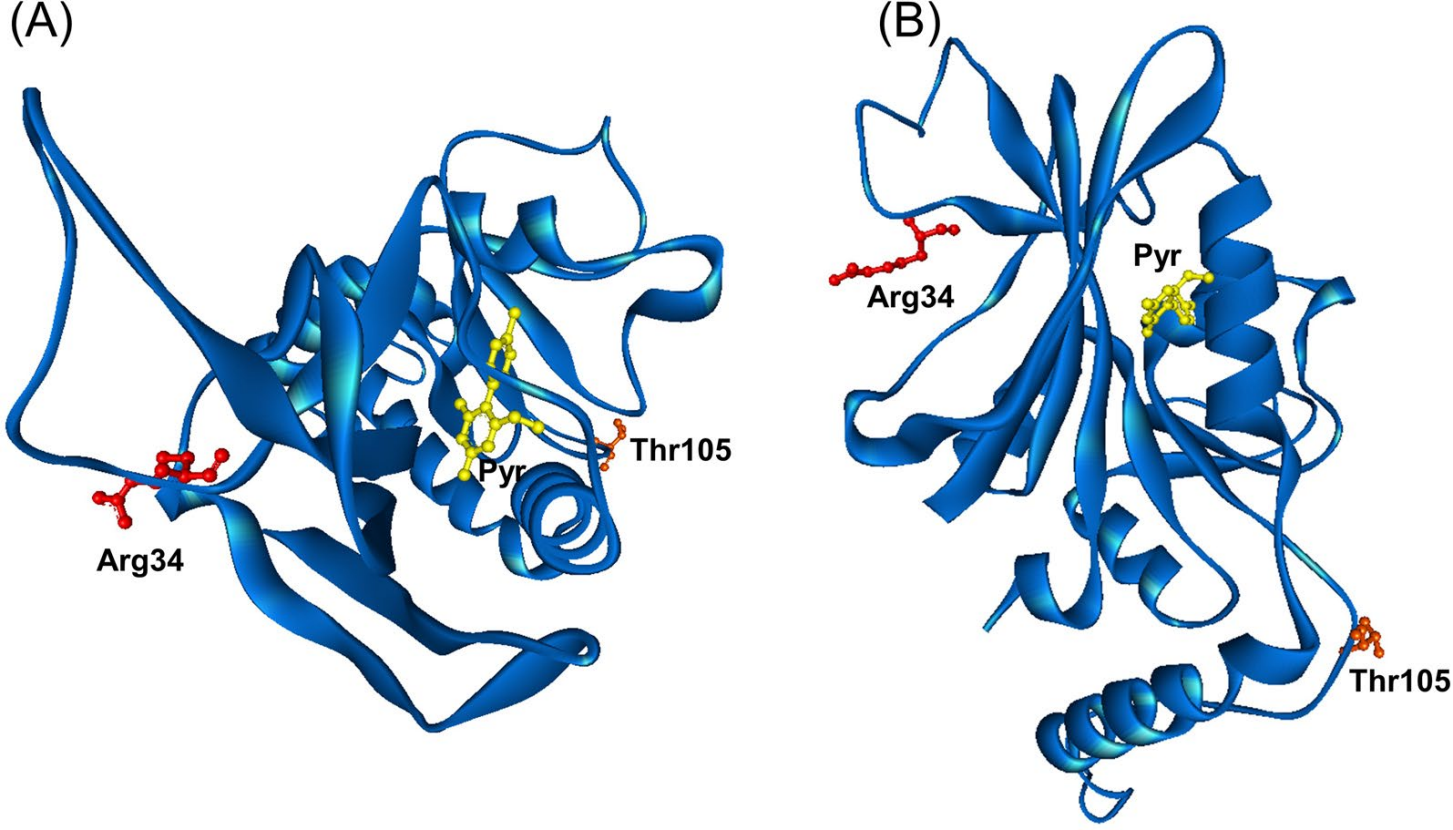
3D structural models of PkDHFR WT in complex with pyrimethamine. **A** Top view and **B** side view. Residues 34 and 105 are depicted in ball and stick colored red and orange, respectively. Pyrimethamine is shown in ball and stick colored yellow

**Fig. 5:**
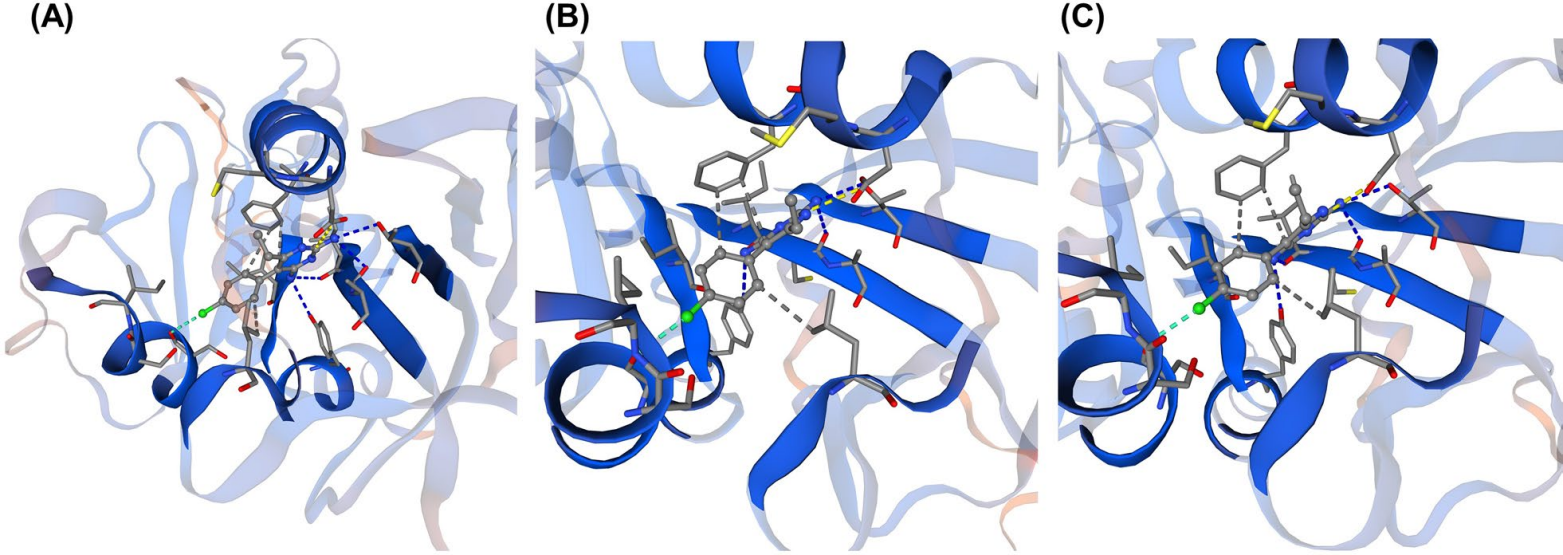
Molecular interactions between PkDHFR **A** WT, **B** Arg34Leu and **C** Thr105 deletion and pyrimethamine inhibitor. Binding analysis suggested that Arg34Leu mutant and Thr105 deletion did not alter binding of DHFR to the inhibitor

Three-dimensional structural models of the two mutants (Arg34Leu and Thr105 deletion) in complex with pyrimethamine was constructed and assessed the effect of these mutations on protein-ligand binding. Neither Arg34 nor Thr105 are part of the binding pocket and are located far from the inhibitor-binding site (Fig. 4). As a consequence, neither the mutation at residue 34 nor the deletion of residue 105 disrupted interactions with the pyrimethamine inhibitor, which was confirmed by binding analysis (Fig. 5).

## Discussion

The six GMS countries have endorsed a malaria elimination plan with the goal of eliminating *P. falciparum* malaria by 2024 and all malaria by 2030 [31]. Although the number of *P. falciparum* and *P. vivax* infections has decreased substantially, the incidence of zoonotic malaria from *P. knowlesi* continues to increase in the GMS subregion [32]. The ongoing increase in *P. knowlesi* incidence presents a major challenge to regional malaria control and prevention activities. *P. knowlesi* infections have been reported in almost all countries in Southeast Asia, and cases have occurred in travelers returning from these countries. However, most infections were reported in Malaysian Borneo [32, 33]. The *P. knowlesi* infections (3.1%) included in this study were found during the Thailand iDES scheme between 2018 and 2020. Furthermore, asymptomatic *P. knowlesi* infections have previously been found at the Thai-Cambodia border [20]. *Plasmodium knowlesi* infection can result in high parasitaemia and death, and the diagnosis should be confirmed by PCR [32]. Therefore, highly specific and sensitive molecular tools and identification are required for malaria detection.

To understand the source of *P. knowlesi* infections in Thailand, microsatellite markers and nucleotide sequences of *pkmsp1* were analysed for comparison with those of *P. knowlesi* isolated from prior reported findings of Thailand [22, 23], Cambodia [20] and Malaysia [24–26], which share borders with Thailand and may be the sources of the *P. knowlesi*. The microsatellite marker and nucleotide sequencing results of *pkmsp1* obtained in this study showed that *P. knowlesi* isolated from southern Thailand were similar to parasites isolated from Malaysia [24–26], suggesting that *P. knowlesi* in southern Thailand may be transmitted from Malaysia. Contrastingly, *P. knowlesi* isolated from eastern Thailand were highly similar to those isolated from Cambodia [20], suggesting this country may be the source of the parasites in that area. The clustering of the parasite lineages is likely to be a result of the migration of macaques, as human-to-human transmission has not been identified and the *Anopheles* vector can only fly a few kilometres. These findings provide information on the source of infection and how *P. knowlesi* malaria may be transmitted.

Molecular clinical and epidemiological studies have clearly shown that specific point mutations in the parasite dihydrofolate reductase gene (*dhfr*) lead to resistance to pyrimethamine. The mutations cause alterations in crucial residues in the active sites of these enzymes, resulting in reduced drug affinity [34–37]. *Plasmodium knowlesi* dihydrofolate reductase (*pkdhfr*) mutations, found in field isolates from many countries, and ex vivo enzyme activity has been the focus of a number of studies. In this study, Arg34Leu and Thr105 deletions were observed in isolates from Thailand. The three-dimensional structural models of the two mutant proteins in complex with pyrimethamine showed that both Arg34 and Thr105 are not part of the binding pocket and are located far from the inhibitor-binding site, suggesting that the mutation at residue 34 and deletion of residue 105 are not associated with pyrimethamine resistance. Other studies have found a number of *pkdhfr* mutations, including Arg34Leu from Sabah, Malaysia, with no signs of positive selection [38]. Moreover, ex vivo enzyme activity has also been studied, but there was no association with antifolate resistance [39]. Anti-malarial drug exposure only occurs in human hosts, and if the transmission of *P. knowlesi* remains zoonotic and there is no selection pressure, the malaria would be unlikely to develop anti-malarial resistance. Although there has yet been no anti-malarial resistance reported in *P. knowlesi*, new anti-malarials should be adopted to counteract emerging anti-malarial resistance in the GMS [40]. These new anti-malarials could aid in resolving anti-malarial resistance issues with other *Plasmodium* species or used in combination to increase anti-malarial efficiency. Furthermore, as monkeys are not treated for malaria, the elimination of *P. knowlesi* is impossible as long as macaques continue to act as zoonotic hosts. This is particularly evident from the experience in Sarawak in Malaysia, where *P. knowlesi* is now almost the only remaining malaria infecting humans [41].

## Conclusions

This study on *P. knowlesi* infections in Thailand demonstrated that the parasites are of the same lineage as *P. knowlesi* isolated in Cambodia and Malaysia and are still sensitive to pyrimethamine. This is useful information for understanding *P. knowlesi* infections in Thailand and for supporting the continuations of malaria elimination programme.

## Data Availability

All data generated or analysed during this study are included in this published article and its Additional files.

## Abbreviations

DNA: Deoxyribonucleic acid
PCR: Polymerase chain reaction
pkmsp1: *P. knowlesi *merozoite surface protein 1 gene
*pkdhfr*: *P. knowesi* dihydrofolate reductase gene

## References

[1] WHO (2019). The “World malaria report 2019” at a glance.

[2] Sudathip P, Saejeng A, Khantikul N, Thongrad T, Kitchakarn S, Sugaram R (2021). Progress and challenges of integrated drug efficacy surveillance for uncomplicated malaria in Thailand. Malar J.

[3] White NJ (2008). *Plasmodium knowlesi*: the fifth human malaria parasite. Clin Infect Dis.

[4] Warren M, Cheong WH, Fredericks HK, Coatney GR (1970). Cycles of jungle malaria in West Malaysia. Am J Trop Med Hyg.

[5] Fong YL, Cadigan FC, Coatney GR (1971). A presumptive case of naturally occurring *Plasmodium knowlesi* malaria in man in Malaysia. Trans R Soc Trop Med Hyg.

[6] Singh B, Kim Sung L, Matusop A, Radhakrishnan A, Shamsul SS, Cox-Singh J (2004). A large focus of naturally acquired *Plasmodium knowlesi* infections in human beings. Lancet.

[7] Singh B, Daneshvar C (2013). Human infections and detection of *Plasmodium knowlesi*. Clin Microbiol Rev.

[8] Yusof R, Lau YL, Mahmud R, Fong MY, Jelip J, Ngian HU (2014). High proportion of knowlesi malaria in recent malaria cases in Malaysia. Malar J.

[9] William T, Jelip J, Menon J, Anderios F, Mohammad R, Awang Mohammad TA (2014). Changing epidemiology of malaria in Sabah, Malaysia: increasing incidence of *Plasmodium knowlesi*. Malar J.

[10] Sermwittayawong N, Singh B, Nishibuchi M, Sawangjaroen N, Vuddhakul V (2012). Human *Plasmodium knowlesi* infection in Ranong province, southwestern border of Thailand. Malar J.

[11] Putaporntip C, Hongsrimuang T, Seethamchai S, Kobasa T, Limkittikul K, Cui L (2009). Differential prevalence of *Plasmodium* infections and cryptic *Plasmodium knowlesi* malaria in humans in Thailand. J Infect Dis.

[12] Ngernna S, Rachaphaew N, Thammapalo S, Prikchoo P, Kaewnah O, Manopwisedjaroen K (2019). Case report: case series of human *Plasmodium knowlesi* infection on the Southern Border of Thailand. Am J Trop Med Hyg.

[13] Jiang N, Chang Q, Sun X, Lu H, Yin J, Zhang Z (2010). Co-infections with *Plasmodium knowlesi* and other malaria parasites, Myanmar. Emerg Infect Dis.

[14] Ghinai I, Cook J, Hla TT, Htet HM, Hall T, Lubis IN (2017). Malaria epidemiology in central Myanmar: identification of a multi-species asymptomatic reservoir of infection. Malar J.

[15] Iwagami M, Nakatsu M, Khattignavong P, Soundala P, Lorphachan L, Keomalaphet S (2018). First case of human infection with *Plasmodium knowlesi* in Laos. PLoS Negl Trop Dis.

[16] Pongvongsa T, Culleton R, Ha H, Thanh L, Phongmany P, Marchand RP (2018). Human infection with *Plasmodium knowlesi* on the Laos-Vietnam border. Trop Med Health.

[17] Van den Eede P, Van HN, Van Overmeir C, Vythilingam I, Duc TN, le Hung X (2008). Human *Plasmodium knowlesi* infections in young children in central Vietnam. Malar J..

[18] Marchand RP, Culleton R, Maeno Y, Quang NT, Nakazawa S (2011). Co-infections of *Plasmodium knowlesi*, *P. falciparum*, and *P. vivax* among humans and *Anopheles dirus* mosquitoes, Southern Vietnam. Emerg Infect Dis..

[19] Khim N, Siv S, Kim S, Mueller T, Fleischmann E, Singh B (2011). *Plasmodium knowlesi* infection in humans, Cambodia, 2007-2010. Emerg Infect Dis.

[20] Imwong M, Madmanee W, Suwannasin K, Kunasol C, Peto TJ, Tripura R (2019). Asymptomatic natural human infections with the simian malaria parasites *Plasmodium cynomolgi* and *Plasmodium knowlesi*. J Infect Dis.

[21] WHO (2018). Malaria rapid diagnostic test performance. Results of WHO product testing of malaria RDTs: round 8 (2016–2018).

[22] Jongwutiwes S, Buppan P, Kosuvin R, Seethamchai S, Pattanawong U, Sirichaisinthop J (2011). *Plasmodium knowlesi* Malaria in humans and macaques, Thailand. Emerg Infect Dis.

[23] Putaporntip C, Thongaree S, Jongwutiwes S (2013). Differential sequence diversity at merozoite surface protein-1 locus of *Plasmodium knowlesi* from humans and macaques in Thailand. Infect Genet Evol.

[24] Divis PC, Singh B, Anderios F, Hisam S, Matusop A, Kocken CH (2015). Admixture in humans of two divergent *Plasmodium knowlesi* populations associated with different macaque host species. PLoS Pathog.

[25] Yap NJ, Goh XT, Koehler AV, William T, Yeo TW, Vythilingam I (2017). Genetic diversity in the C-terminus of merozoite surface protein 1 among *Plasmodium knowlesi* isolates from Selangor and Sabah Borneo, Malaysia. Infect Genet Evol.

[26] Yap NJ, Vythilingam I, Hoh BP, Goh XT, Muslim A, Ngui R (2018). Genetic polymorphism and natural selection in the C-terminal 42 kDa region of merozoite surface protein-1 (MSP-1) among *Plasmodium knowlesi* samples from Malaysia. Parasit Vectors.

[27] Snounou G, Viriyakosol S, Zhu XP, Jarra W, Pinheiro L, do Rosario VE (1993). High sensitivity of detection of human malaria parasites by the use of nested polymerase chain reaction. Mol Biochem Parasitol.

[28] Waterhouse A, Bertoni M, Bienert S, Studer G, Tauriello G, Gumienny R (2018). SWISS-MODEL: homology modelling of protein structures and complexes. Nucleic Acids Res.

[29] Morris AL, MacArthur MW, Hutchinson EG, Thornton JM (1992). Stereochemical quality of protein structure coordinates. Proteins.

[30] Imwong M, Pukrittakayamee S, Looareesuwan S, Pasvol G, Poirreiz J, White NJ (2001). Association of genetic mutations in *Plasmodium vivax* dhfr with resistance to sulfadoxine-pyrimethamine: geographical and clinical correlates. Antimicrob Agents Chemother.

[31] WHO (2015). Strategy for malaria elimination in the Greater Mekong Subregion: 2015–2030.

[32] WHO (2017). Expert consultation on *Plasmodium knowlesi* malaria to guide malaria elimination strategies.

[33] Anstey NM, Grigg MJ (2019). Zoonotic malaria: the better you look, the more you find. J Infect Dis.

[34] Foote SJ, Cowman AF (1994). The mode of action and the mechanism of resistance to antimalarial drugs. Acta Trop.

[35] Matthews DA, Alden RA, Bolin JT, Freer ST, Hamlin R, Xuong N (1977). Dihydrofolate reductase: x-ray structure of the binary complex with methotrexate. Science.

[36] Peterson DS, Walliker D, Wellems TE (1988). Evidence that a point mutation in dihydrofolate reductase-thymidylate synthase confers resistance to pyrimethamine in falciparum malaria. Proc Natl Acad Sci USA.

[37] Volz KW, Matthews DA, Alden RA, Freer ST, Hansch C, Kaufman BT (1982). Crystal structure of avian dihydrofolate reductase containing phenyltriazine and NADPH. J Biol Chem.

[38] Grigg MJ, Barber BE, Marfurt J, Imwong M, William T, Bird E (2016). Dihydrofolate-reductase mutations in *Plasmodium knowlesi* appear unrelated to selective drug pressure from putative human-to-human transmission in Sabah, Malaysia. PLoS ONE..

[39] Ittarat W, Pornthanakasem W, Mungthin M, Suwandittakul N, Leelayoova S, Tarnchompoo B (2018). Characterization of *Plasmodium knowlesi* dihydrofolate reductase-thymidylate synthase and sensitivity to antifolates. Parasitol Int.

[40] WHO (2017). Status report on artemisinin and ACT resistance.

[41] Feachem RGA, Chen I, Akbari O, Bertozzi-Villa A, Bhatt S, Binka F (2019). Malaria eradication within a generation: ambitious, achievable, and necessary. Lancet.

